# Patterns of family–school environment influence depressive symptoms among adolescents in China: the mediating role of resilience

**DOI:** 10.3389/fpsyt.2025.1717022

**Published:** 2025-11-21

**Authors:** Suqun Liao, Dongfang Wang

**Affiliations:** 1 College of Educational Sciences, Shaoguan University, Shaoguan, China; 2 School of Psychology, Centre for Studies of Psychological Applications, Guangdong Key Laboratory of Mental Health and Cognitive Science, Ministry of Education Key Laboratory of Brain Cognition and Educational Science, Guangdong Emergency Response Technology Research Center for Psychological Assistance in Emergencies, South China Normal University, Guangzhou, China

**Keywords:** family function, school climate, depressive symptoms, resilience, latent profile analysis

## Abstract

**Background:**

Previous studies have shown that both family and school environments are significantly associated with depressive symptoms in adolescents. However, it remains unclear how specific patterns of the family–school environment influence later depressive symptoms and what mechanisms may explain this relationship.

**Methods:**

A total of 11860 adolescents completed web-based surveys during two periods: 21 April to 12 May 2021 (Time 1, T1) and 17 to 26 December 2021 (Time 2, T2). Participants reported on sample characteristics, family function, school climate, resilience, depressive symptoms, and negative life events. Latent profile analysis was used to identify profiles of family–school environment, and mediation analyses were conducted to examine whether resilience mediates the associations between these environmental profiles and depressive symptoms.

**Results:**

Four distinct family–school environment profiles were identified: (1) family dysfunction–poor school climate (32.2%), (2) good family function–good school climate (27.8%), (3) good family function–poor school climate (26.6%), and (4) family dysfunction–good school climate (13.4%). Compared with the “good family function–good school climate” group, participants in the other three profiles showed higher levels of depressive symptoms at T2, mediated by lower levels of resilience.

**Conclusions:**

These findings highlight the importance of monitoring adolescents’ perceptions of their family and school environments in early depression screening. Timely and targeted interventions aimed at enhancing resilience may be beneficial for adolescents in high-risk environmental profiles.

## Introduction

Depressive symptoms have emerged as a critical global public-health challenge, ranking as the foremost driver of health-related disability and a principal contributor to the worldwide burden of disease (1, 2). Among adolescents, these symptoms are one of the strongest predictors of suicidality (3, 4). Depressive symptoms are both highly prevalent and are increasing annually among adolescents (5). One meta-analysis found that about 34% of individuals aged 10 to 19 years manifest elevated depressive symptoms, a prevalence that exceeds all previously documented estimates for the 18–25 age group (6). In China, the prevalence of adolescent depressive symptoms ranges from 4% to 41% (7). Given the high prevalence and adverse consequences of depressive symptoms among adolescents, it is imperative to identify modifiable and predictive factors to inform prevention efforts.

According to ecological systems theory, individual psychological development is the result of the interaction between environmental and individual factors (8). In terms of environmental factors, the family and school are key developmental contexts for children and adolescents and have been shown to be closely linked to their depressive symptoms (9, 10). Among these, family function can be understood as the capacity of the family to operate as a cohesive unit in order to meet the basic needs of its members. It encompasses the emotional bonds among family members, family rules, family communication, and the effectiveness with which the family responds to external events (11). Empirical evidence indicates that sound family function serves as a protective factor against depressive symptoms in adolescents (12), whereas family dysfunction heightens depressive risk by amplifying environmental stress (13). Likewise, there is a well-established link between school climate and adolescent depressive symptoms (14, 15). School climate herein is conceptualized as the perceived quality and character of school life, which can reflect the norms, values, and expectations of a school (16). Cross-sectional (17) and longitudinal studies (18) have consistently documented an inverse association between positive school climate and adolescent depressive symptoms.

Although the family and the school constitute the primary environmental contexts for school-aged adolescents, most existing studies have examined risk factors in isolation, focusing exclusively on either the family or the school setting. This study extends a limited research base by simultaneously testing effects of family influences (i.e., family function) and school influences (i.e., school climate) on adolescent depressive symptoms. Moreover, by adopting person-oriented methods such as latent class analysis (LCA) and latent profile analysis (LPA), researchers can anchor their investigations in adolescents’ real-life developmental contexts. These approaches allow for the integration of multiple family- and school-level indicators to uncover latent subgroups with highly similar family–school experiential profiles, thereby mapping the heterogeneous patterns of adolescents’ family–school environments with greater nuance and depth (19).

Resilience, as a modifiable factor and an important mediator of depressive symptoms, is further examined in this study. Grounded in the resilience framework, resilience operates as a dynamic process that mediates between hardship and growth, enabling individuals to advance in the face of adversity (20). Empirical findings have shown that family function is positively correlated with resilience (21). Research also indicates a significant correlation between school climate and resilience, and the better the school climate, the higher the resilience of adolescents (22). Moreover, adolescents with higher resilience are less likely to exhibit depressive symptoms (23). Therefore, resilience may mediate both the link between family function and depressive symptoms and the link between school climate and depressive symptoms. A series of studies have explored the mediating role of resilience in the relationship between family–school environmental factors and mental health among adolescents. For instance, a previous study showed that resilience mediates the relationship between family function and emotional behavior problems among adolescents (24). Our team’s earlier research also revealed that resilience serves as a mediator between school climate and psychotic-like experiences (25).

In this study, two waves of surveys were conducted among Chinese adolescents, with a 6-month interval between them, to pursue three primary aims. First, using LPA, we identified distinct family–school environment profiles by integrating multiple indicators of family function and school climate at baseline. Second, we examined whether these profiles were differentially linked to subsequent depressive symptoms. Finally, we tested whether resilience mediated the associations between family–school environment profiles and subsequent depressive symptoms.

## Methods

### Study design and population

The data for this study were obtained from a semester-based adolescent mental health survey conducted in Bao’an District, Shenzhen. This study is based on a two-wave longitudinal dataset with a repeated measures design. The detailed sampling and data-collection procedures have been described elsewhere (25). In brief, we conducted online assessments of 51568 and 47243 Grade 7 and Grade 8 students from 21 April to 12 May 2021 (Time 1, T1) and six months later from 17 to 26 December 2021 (Time 2, T2), respectively. Participants were excluded due to incorrect identity information (e.g., incorrect student number), excessively short response times (e.g., less than 5 min), inconsistent responses (e.g., fill in the same answer consecutively), or a history of mental-health disorders. After exclusion, 50625 participants remained at T1 and 42409 at T2. Through data integration, a total of 11860 junior high school students participated in both web-based surveys and provided complete and valid data on all measures. Compared to those lost to follow-up, these students were slightly younger (13.40 ± 0.76 *vs*.13.49 ± 0.79, t=-1.21, p <0.001), and a higher proportion were female (47.7% *vs*. 44.5%, χ^2^ = 33.17, p <0.001) or in grade 7 (59.3% *vs*. 52.3%, χ^2^ = 155.63, p <0.001).

Before the survey began, school teachers sent invitation letters to students and their parents and obtained informed consent from both. The survey was conducted entirely through the “Survey Star” platform; all participants could access the electronic questionnaire by scanning a QR code with their mobile phones and complete it at their convenience. At any point during the survey, participants could stop or withdraw if they felt uncomfortable. We also opened a psychological hotline to provide free mental-health support for any student in need.

Our study was carried out in accordance with the Helsinki Declaration as revised 1989 and approved by the Human Research Ethics Committee of School of Psychology of South China Normal University (Ethics No. SCNU- PSY- 2021- 094).

### Measuring instruments

#### Family function

Family function at baseline was evaluated with the family APGAR index, a five-item instrument that captures five dimensions: adaptability, partnership, growth, affection, and resolve (26). Each item is ranked on a 3-point Likert scale, ranging from 0 (hardly ever) to 2 (almost always). The total score ranges from 0 to 10, with higher scores reflecting greater family function. A score of 0~3 denotes severe family dysfunction, 4~6 moderate family dysfunction, and 7~10 good family function. This scale has high internal consistency in the current study (Cronbach’ α=0.92).

#### School climate

School climate at baseline was measured with the 2016 Version of Delaware School Climate Scale-Student (DSCS-S) (27). The 31-item scale evaluates seven dimensions: teacher-student relations (5 items), student-student relations (5 items), student engagement (6 items), clarity of expectations (4 items), fairness of rules (4 items), school safety (3 items), and bullying (4 items, *reverse scoring*). Each item is scored on a 4-point Likert scale, ranging from 1 (completely disagree) to 4 (completely agree). The higher the total score, the better the school climate. The Chinese version of DSCS-S has satisfactory psychometric properties (28). The Cronbach’ α in this study was 0.96.

#### Resilience

Resilience at baseline was assessed with the 10-item Connor-Davidson Resilience Scale (CD-RISC-10) (29). Responses are made on a scale from 0 (never) to 4 (almost always), with higher total scores reflecting greater resilience. Psychometric properties of the Chinese version of the CD-RISC-10 have been described elsewhere (30). In this sample, the scale demonstrated excellent internal consistency (Cronbach’s α = 0.95).

#### Depressive symptoms

Depressive symptoms was measured with the 9-item Patient Health Questionnaire (PHQ-9) (31). Each item is rated on a 4-point Likert-type scale ranging from 0 (not at all) to 3 (almost every day). Higher total scores indicate greater severity of depressive symptoms experienced over the past two weeks. The Chinese version of the PHQ-9 has demonstrated satisfactory applicability (32). In this study, Cronbach’ α was 0.91 at T1 and 0.92 at T2.

#### Covariates

Sample characteristics were collected via self-report and included sex, age, grade, ethnicity, parental marital status, single child status, parental education, chronic physical illness, and family history of mental disorder.

Negative life events experienced by adolescents between the two surveys were considered as an important covariate. The Adolescent Self-Rating Life Events Checklist (ASLEC), employed at T2, assessed negative life events over the previous six months (33). The 27-item scale comprises six factors: interpersonal conflict, academic pressure, punishment, personal loss, physical health problems, and others. Each item is rated from 1 (not at all) to 5 (extremely severe), with higher total scores indicating greater perceived negative stress. Cronbach’s α in the present study was 0.96.

### Statistical analysis

SPSS 24.0 and Mplus 7.4 were used for all statistical analyses. Using the standardized scores on the five dimensions of family functioning and the seven dimensions of school climate as indicators, latent profile analysis (LPA) was employed to determine the most likely number of family–school environment profiles. To identify the optimal number of latent profiles, we fitted a series of models beginning with a one-class solution and incrementally adding classes. Model fit was evaluated with the Akaike information criterion (AIC), Bayesian information criterion (BIC), sample-size-adjusted BIC (aBIC), entropy, the bootstrap likelihood ratio test (BLRT), and the Lo–Mendell–Rubin likelihood ratio test (LMR-LRT). Preference was given to models exhibiting lower AIC, BIC, and aBIC values (34), higher Entropy value (35), and statistically significant BLRT and LMR-LRT results (*p* < 0.05) (36). To prevent spurious profiles and over-extraction, we assessed all profiles and ensured each profile comprised at least 5% of the samples (37).

An ANOVA test was employed to examine between-group differences in scores on family function, school climate, resilience, and depressive symptoms. Spearman’s correlations were conducted to assess the associations among these variables. Finally, we conducted a mediation analysis with 5000 bootstrap iterations to construct 95% confidence intervals (CIs) and test whether resilience (T1) mediated the relationship between family–school environment profiles (T1) and subsequent depressive symptoms (T2). Following Hayes’ recommendations (38), Model 4 of PROCESS 3.3 was specified: profiles of family–school environment (T1) were incorporated as the predictor, with resilience (T1) as the mediator and depressive symptoms (T2) as the outcome. All sample characteristics (i.e., sex, age, grade, ethnicity, parental marital status, single child status, parental education, chronic physical illness, and family history of mental disorder), baseline depressive symptoms, and negative life events (T2) were included as potential covariates. The categorical predictor was handled via indicator (dummy) coding (39), while continuous variables were standardized. This multi-categorical mediation strategy allowed us to estimate the total, direct, and indirect effects of each family–school environment profile (T1) on later depressive symptoms (T2) through mediating pathway of resilience (T1).

## Results

### Description of the sample and correlations among main variables

Among the participants, 52.3% are boys, with baseline mean age of 13.40 years (SD = 0.76). The proportion of participants in Grade 7 is slightly higher than that in Grade 8 (59.3% *vs*. 40.7%). A majority of the participants were ethnicity Han (N = 11391, 96.0%) and one in five adolescents is an only child (N = 2402, 20.3%). **Table 1** shows detailed sample characteristic variables.

**Table 1:** Baseline sample characteristics of participants (N=11860).

Characteristics		N(%)
Sex	Male	6202(52.3)
	Female	5658(47.7)
Age [years, M(SD)]		13.40(0.76)
Grade	7th	7028(59.3)
	8th	4832(40.7)
Ethnicity	Han ^a^	11391(96.0)
	Others	469(4.0)
Parental marital status	Married	11252(94.9)
	Not current married ^b^	608(5.1)
Single child status	Yes	2402(20.3)
Father’s education	Junior high school or below	3658(30.8)
	Senior high school	3456(29.1)
	College or above	4746(40.1)
Mother’s education	Junior high school or below	4330(36.5)
	Senior high school	3320(28.0)
	College or above	4210(35.5)
Chronic physical illness ^c^	Yes	443(3.7)
Family history of mental disorder	Yes	92(0.8)

a Han is the major ethnic group in China.

b Not current married included separated, divorced and widowed.

c Chronic physical conditions referred to having at least one of arthritis, angina, asthma, diabetes, visual impairment, or hearing problems

Depressive symptoms (T2) was significantly negatively correlated with family function (T1) (r = -0.25), school climate (T1) (r = -0.22), and resilience (T1) (r = -0.30). Additionally, resilience (T1) showed a significant positive association with both family function (T1) (r = 0.42), school climate (T1) (r = 0.43), all *p* < 0.001.

### Patterns of family-school environment

The results of the LPA are presented in **Table 2**. The BLRT and VLMR-LRT both yielded statistically significant results for the three- and four-class solutions, thereby justifying the retention of more than two latent classes. Furthermore, the four-class model demonstrated superior fit relative to the three-class model, as evidenced by its consistently lower AIC, BIC, and aBIC values. The four-class model further exhibited a higher Entropy value of 0.960, reflecting superior classification accuracy. The five-class model was excluded because the smallest class accounted for less than 5% of the total sample. Hence, the four-class model was established as optimal, with samples assigned to each latent category at an average probability of 96.3%~98.2%.

**Table 2:** Fit indices for latent profile analyses.

Class	AIC	BIC	aBIC	Entropy	BLRT (p)	LMR-LRT (p)	Smallest class (%)
1	403922.66	404099.81	404023.54				
2	339627.82	339900.91	339783.33	0.971	<0.001	0.274	40.7
3	320943.70	321312.74	321153.85	0.951	<0.001	<0.001	26.6
4	**304730.02**	**305195.02**	**304994.82**	**0.960**	**<0.001**	**<0.001**	**13.4**
5	292420.90	292981.85	292740.33	0.967	<0.001	0.003	1.7

Bold indicates best fit. Entropy and value of p for BLRT, aLMR, and VLMR are not applicable to a one-class solution. AIC, Akaike information criterion; BIC, Bayesian information criterion; aBIC, sample-size-adjusted Bayesian information criterion; BLRT, bootstrap likelihood ratio test; LMR-LRT, Lo-Mendell-Rubin Likelihood Ratio Test.

**Figure 1:**
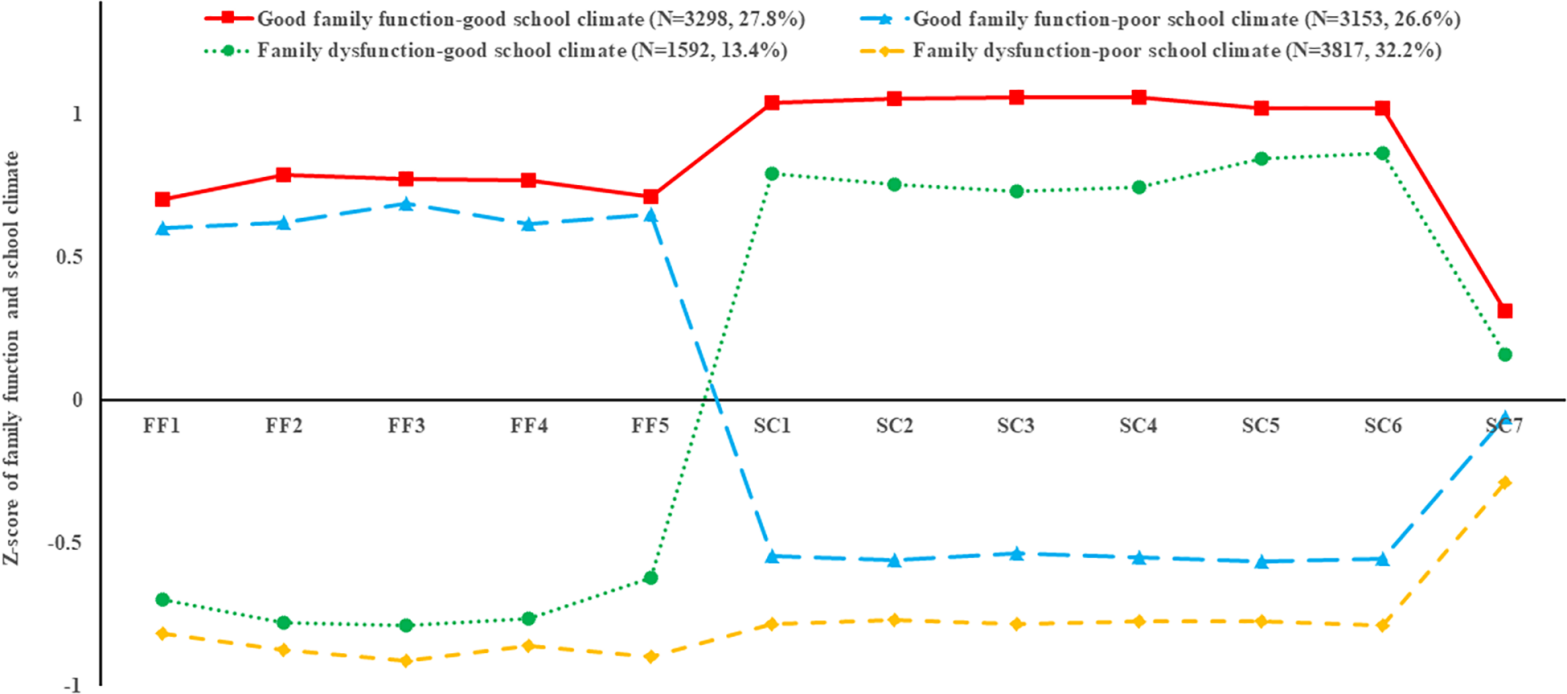
Four latent profiles of family-school environment; FF1: Adaptation, FF2: Partnership, FF3: Growth, FF4: Affect, FF5: Resolve, SC1: Teacher-student relations, SC2: Student-student relations, SC3: Student engagement, SC4: Clarity of expectations, SC5: Fairness of rules, SC6: School safety, SC6: Bullying (reverse scoring).

Four latent profiles of family-school environment are shown in **Figure 1**. The four profiles were as follows (1): good family function-good school climate group (Profile 1; 27.8%, N = 3298), characterized by the highest scores on family function and school climate (2); good family function-poor school climate group (Profile 2; 26.6%, N = 3153), characterized by having high-average scores on family function and low-average scores on school climate (3); family dysfunction-good school climate group (Profile 3; 13.4%, N = 1592), characterized by low-average scores on family function and high-average scores on school climate (4); family dysfunction-poor school climate group (Profile 4; 32.2%, N = 3817), characterized by the lowest scores on family function and school climate. Descriptive statistics of family function, school climate, resilience, and depressive symptoms in different family-school environment profiles were illustrated in **Table 3**.

**Table 3:** Family function, school climate, resilience, and depressive symptoms across profiles [M(SD)].

	Total	1. Good family function-good school climate (27.8%)	2. Good family function-poor school climate (26.6%)	3. Family dysfunction-good school climate (13.4%)	4. Family dysfunction-poor school climate (32.2%)	Pos hoc
Family function (T1)	7.06(2.82)	9.49(0.88)	9.12(1.09)	4.70(1.70)	4.25(1.76)	1>2>3>4
Adaptation,	1.36(0.65)	1.82(0.41)	1.76(0.45)	0.92(0.48)	0.84(0.47)	1>2>3>4
Partnership	1.35(0.68)	1.88(0.33)	1.77(0.43)	0.82(0.48)	0.76(0.47)	1>2>3>4
Growth	1.47(0.63)	1.96(0.19)	1.91(0.29)	0.97(0.50)	0.89(0.48)	1>2>3>4
Affect	1.35(0.68)	1.87(0.35)	1.77(0.43)	0.84(0.50)	0.78(0.47)	1>2>3>4
Resolve	1.53(0.60)	1.96(0.20)	1.92(0.27)	1.15(0.56)	0.98(0.51)	1>2>3>4
School climate (T1)	101.94(13.71)	117.43(5.25)	94.10(5.97)	113.35(6.49)	90.27(8.04)	1>3>5>4
Teacher-student relations	16.83(2.58)	19.51(1.02)	15.42(1.49)	18.88(1.43)	14.81(1.84)	1>3>5>4
Student-student relations	16.63(2.62)	19.40(1.17)	15.18(1.37)	18.61(1.72)	14.63(1.82)	1>3>5>4
Student engagement	19.64(3.00)	22.83(1.58)	18.04(1.46)	21.84(1.95)	17.29(2.00)	1>3>5>4
Clarity of expectations	13.16(2.05)	15.34(1.04)	12.03(1.03)	14.69(1.36)	11.57(1.37)	1>3>5>4
Fairness of rules	13.44(2.15)	15.64(0.80)	12.23(1.18)	15.26(1.13)	11.77(1.59)	1>3>5>4
School safety	10.18(1.59)	11.81(0.54)	9.03(0.91)	11.56(0.77)	8.93(1.18)	1>3>5>4
Bullying (reverse scoring)	12.05(2.71)	12.90(3.25)	11.89(2.09)	12.49(3.16)	11.27(2.16)	1>3>5>4
Resilience (T1)	27.20(9.04)	32.80(7.58)	27.66(7.47)	26.92(9.04)	22.08(8.41)	1>2>3>4
Depressive symptoms (T1)	4.66(4.86)	2.58(3.53)	3.75(3.87)	5.36(5.19)	6.92(5.42)	4>3>2>1
Depressive symptoms (T2)	4.66(4.88)	3.14(4.09)	4.34(4.50)	5.01(5.11)	6.09(5.27)	4>3>2>1

### The mediating role of resilience

**Figure 2:**
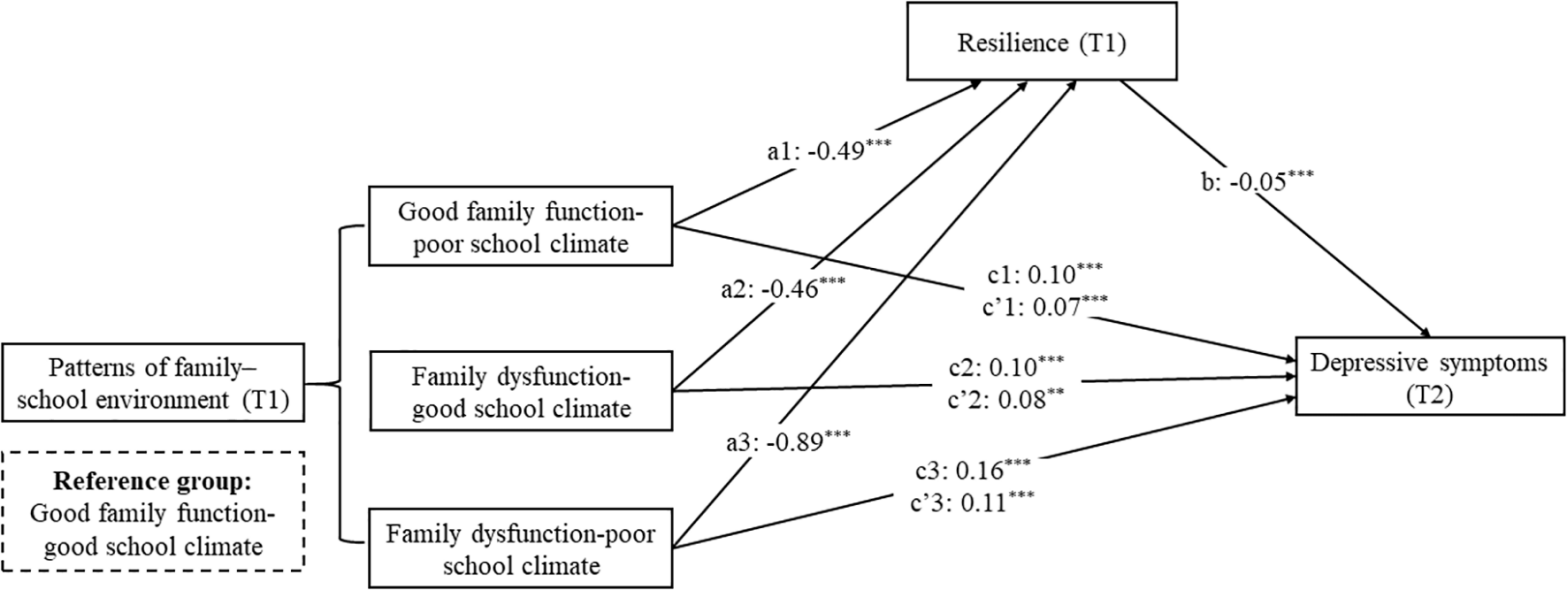
Path analysis of the association between patterns of family–school environment (T1) and depressive symptoms (T2) through resilience. This mediating model adjusted for sample characteristics (**Table 1**), depressive symptoms (T1), and negative life events (T2). **p<0.01, ***p<0.001.

The mediation models are shown in **Figure 2**, and the 95% confidence intervals (CIs) from the bias-corrected bootstrap analyses are provided in **Table 4**. Compared with the good family function–good school climate group, participants in the good family function–poor school climate group (a1 = -0.49), the family dysfunction–good school climate group (a2 = -0.46), and the family dysfunction–poor school climate group (a3 = -0.89) reported significantly lower resilience at T1. Higher resilience at T1 predicted a lower likelihood of depressive symptoms at T2 (b = -0.05). The relative indirect effects were 0.03 (a1 × b) for the good family function–poor school climate group, 0.02 (a2 × b) for family dysfunction–good school climate group, and 0.05 (a3 × b) for the family dysfunction–poor school climate group. Mediation analyses also revealed a significant relative direct effect of the good family function–poor school climate group (c’1 = 0.07), the family dysfunction–good school climate group (c’2 = 0.08), and the family dysfunction–poor school climate group (c’3 = 0.11) (compared to reference group) on depressive symptoms (T2). These findings indicate that, relative to the good family function–good school climate group, participants in the other three groups were more likely to show increased depressive symptoms six months later, partially mediated by a decrease in resilience at T1.

**Table 4:** Results of the mediation analysis.

Relative effects of specific paths	*β*		S.E.	95% CI
Good family function-poor school climate (T1)→ Resilience (T1)	-0.49		0.02	-0.53,-0.45
Family dysfunction-good school climate (T1)→ Resilience (T1)	-0.46		0.03	-0.51,-0.41
Family dysfunction-poor school climate (T1)→ Resilience (T1)	-0.89		0.02	-0.94,-0.85
Resilience (T1)→ Depressive symptoms (T2)	-0.05		0.01	-0.07,-0.03
Good family function-poor school climate (T1)→ Depressive symptoms (T2)	0.07		0.02	0.03,0.11
Family dysfunction-good school climate (T1)→ Depressive symptoms (T2)	0.08		0.03	0.03,0.13
Family dysfunction-poor school climate (T1)→ Depressive symptoms (T2)	0.11		0.02	0.06,0.15
Relative indirect effects	Effect	Bootstrap SE	Bootstrap 95% CI	|ai×b/ci|
Good family function-poor school climate (T1)→ Resilience (T1)→ Depressive symptoms (T2)	0.03	0.005	0.02,0.04	0.03/0.10=30.0%
Family dysfunction-good school climate (T1)→ Resilience (T1)→ Depressive symptoms (T2)	0.02	0.005	0.01,0.03	0.02/0.10=20.0%
Family dysfunction-poor school climate (T1)→ Resilience (T1)→ Depressive symptoms (T2)	0.05	0.01	0.03,0.06	0.05/0.16=31.3%

Adjusting for sample characteristics (**Table 1**), depressive symptoms (T1), and negative life events (T2).

## Discussion

To our knowledge, this study is the first to uncover population heterogeneity in the family–school environment. As expected, the LPA identified four distinct profiles: “family dysfunction–poor school climate” (the largest group, 32.2%), followed by “good family function–good school climate” (27.8%), “good family function–poor school climate” (26.6%), and “family dysfunction–good school climate” (13.4%). Previous research suggests that family and school environments are mutually influential (40). However, from a person-centered perspective, adolescents’ perceived family–school environments may be markedly heterogeneous. Consequently, more nuanced analyses are needed when examining how these environments affect the individual.

Previous studies have shown that the family-school environment is closely associated with depressive symptoms in adolescents (9, 10). The present research extends these findings by demonstrating a direct link between patterns of the family–school environment and adolescent depressive symptoms. Specifically, compared with the “good family function–good school climate” group, adolescents in the other three profiles faced a higher subsequent risk of depressive symptoms. Among them, adolescents in the “family dysfunction–poor school climate” group reported the most severe depressive symptoms six months later. Previous investigations have consistently identified family function as a robust predictor of adolescent depressive symptoms (12, 13). Adolescents reared in highly dysfunctional families exhibit elevated vulnerability to negative self-cognitions, which have been empirically identified as a critical proximal mechanism precipitating the onset of depressive symptomatology (41, 42). Similarly, a negative association between school climate and depressive symptoms also has been supported in previous studies (17, 18). When students perceive the school climate as unsupportive, they struggle to feel emotionally safe and become less willing to seek help from teachers when facing difficulties, which in turn heightens their risk of depressive symptoms (43).

This study found that resilience mediates the relationship between patterns of the family–school environment and depressive symptoms. Specifically, compared with the “good family function-good school climate” group, adolescents in the other three family-school environment patterns exhibited more depressive symptoms through decreased resilience. Rutter proposed that resilience develops protective mechanisms that can mitigate and counteract the impact of risk factors and negative events (44). These mechanisms involve harnessing constructive strengths from internal personal sources and leveraging support from external environmental resources to break down or even eliminate negative influences. For example, when students perceive better family functioning, they experience more harmonious family relationships and higher-quality parent–child communication, which can significantly foster positive developmental attributes (i.e., resilience) (45). Meanwhile, a positive school climate can enhance students’ sense of belonging to the campus and provide them with greater psychological support (46), thereby further promoting their resilience. Furthermore, ample evidence supports that resilience is a protective factor against poor mental health, with higher resilience helping to alleviate depressive symptoms (23, 47). Hence, this study confirms that a negative family–school environment diminishes the level of resilience, which in turn increases the likelihood of depressive symptoms.

This study highlights the significant impact of the family–school environment on adolescent depressive symptoms. In clinical and educational practice, at-risk adolescents can be identified early by assessing their perceptions of family function and school climate, especially students who experience both family dysfunction and perceive a poor school climate. Meanwhile, the family- and school-based interventions also show promise for alleviating emotional disorders in children and adolescents (48, 49). Given the mediating role of resilience, measures such as universal resilience-focused interventions (50) aimed at enhancing adolescents’ resilience may be effective in alleviating their depressive symptoms. Overall, this study suggests that educators and clinical practitioners may tailor intervention programs according to different environmental characteristics. For instance, for groups with good family function but a poor school climate, priority could be given to improving the school environment. For those with family dysfunction but a positive school climate, family-based interventions should be emphasized. In cases where both family function and school climate are poor, a combined home–school intervention supplemented with resilience training is recommended.

The strengths of this study include its large sample, longitudinal design, and person-centered perspective. Nevertheless, several limitations need to be acknowledged. First, depressive symptoms in the present study were assessed via self-report, and participants may have introduced reporting bias due to social-desirability effects. Future studies may consider employing multi-informant assessments (e.g., parental, teacher, and clinical evaluations) or incorporating objective physiological indicators (e.g., salivary cortisol, heart rate variability). Second, all participants were recruited from a single district in Shenzhen and did not include rural adolescents. Meanwhile, due to factors such as delayed school notifications about the survey, scheduling conflicts with major school events (e.g., midterm exams and sports meets), the attrition rate during follow-up was relatively high. Therefore, the generalizability of the findings to this population should be interpreted with caution. Third, the six-month interval between the two time points may have captured only the short-term effects of family–school environments on depressive symptoms. Future studies should incorporate additional follow-up assessments to explore the long-term impact of these environments on adolescent mental health.

## Conclusion

Family and school environments are closely linked to adolescents’ mental health. There has been a dearth of research on patterns of the family–school environment and their effects on depressive symptoms, as well as on the potential mediating mechanisms. This study fills that gap by exploring how profiles of the family–school environment predict subsequent depressive symptoms through the mediation of resilience. Four distinct profiles emerged among Chinese adolescents: family dysfunction–poor school climate, good family function–good school climate, good family function–poor school climate, and family dysfunction–good school climate. Compared with the good family function–good school climate group, participants in the other three profiles exhibited higher levels of depressive symptoms via reduced resilience. Therefore, the findings underscore the necessity of closely monitoring adolescents’ perceptions of their family and school environments during early depression screening and of delivering timely, targeted interventions to high-risk groups to enhance their resilience.

## Data Availability

The raw data supporting the conclusions of this article will be made available by the authors, without undue reservation.
